# Mineral trioxide aggregate a boon for prevention of external root resorption

**DOI:** 10.11604/pamj.2022.42.119.34813

**Published:** 2022-06-14

**Authors:** Akash Jitender Sibal, Akshay Ajay Jaiswal

**Affiliations:** 1 Department of Conservative Dentistry and Endodontics Sharad Pawar Dental College and Hospital, Datta Meghe Institute of Medical Sciences (Deemed to be University), Wardha, India

**Keywords:** Root resorption, distal root, mandibular 1^st^ molar

## Image in medicine

External root resorption is a lytic process occurring in the cementum and/or dentine of the roots of teeth. Although till date the predisposing factor is yet to be discovered, the suspected causes are considered to be peri radicular inflammation due to trauma, granuloma, central jaw tumors, excessive forces, impaction of teeth, and systemic diseases. A 23-year-old male reported to the department of conservative dentistry and endodontics with a chief complaint of pain and food lodgement in lower left mandibular region since 5-6 months. On clinical examination, grossly decayed tooth (#36) which was tender on vertical percussion and was not associated with any mobility. Intraoral periapical radiographic revealed external root resorption in distal root, which was concomitant with mild bone loss. Taking into account the extent and the severity of the resorption the treatment course was planned that included Root Canal Treatment. The distal and two mesial canals were obturated using mineral trioxide aggregate and gutta-percha respectively. After completion of obturation, it was followed by post endodontic composite restoration. The patient was followed-up after 1 year and the tooth was functional without sensitivity to percussion or palpation. Tooth showed normal physiologic mobility and no periodontal pockets on probing. Radiographic findings revealed no periapical changes and no further progression of External root resorption.

**Figure 1:**
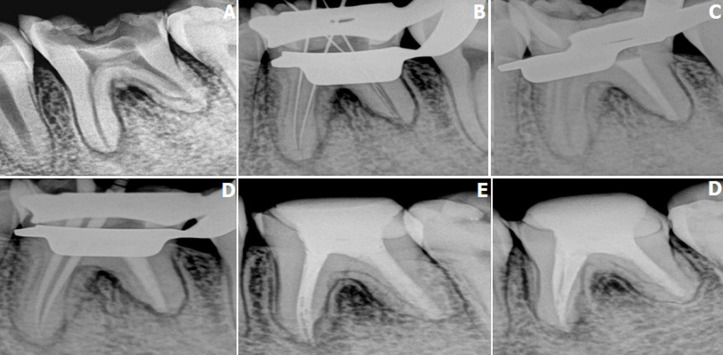
pre-operative image showing external root resorption with distal root (A); working length determination (B); complete obturation using MTA in distal canal (C); evaluation of master cone fit in mesial canal (D); obturation with gutta percha in mesial canal (E); patient follow-up after 1 year showed no degenerative changes (F)

